# Combined Computational Systems Biology and Computational Neuroscience Approaches Help Develop of Future “Cognitive Developmental Robotics”

**DOI:** 10.3389/fnbot.2017.00063

**Published:** 2017-11-15

**Authors:** Faramarz Faghihi, Ahmed A. Moustafa

**Affiliations:** ^1^Department for Cognitive Modeling, Institute for Cognitive and Brain Sciences, Shahid Beheshti University, Tehran, Iran; ^2^School of Social Sciences and Psychology and Marcs Institute for Brain and Behavior, Western Sydney University, Sydney, NSW, Australia

**Keywords:** neural networks (computer), spiking neurons, intelligent systems, cognitive developmental robotics, computational systems biology

## Brain, natural intelligence, and cognitive developmental robotics

Understanding cognitive functions and mechanisms of development in animals is essential for the future generation of more intelligent systems (Hirel et al., 2011; Hassabis et al., 2017). In traditional robotics the robots perform predefined tasks in a fixed environment. However, the field of modern robotics is seeking approaches to develop artificial systems to execute tasks in less predefined dynamic environments. Such robotic systems should learn from information extracted from the environment to demonstrate actions like natural intelligence (Matarić, 1998). However, such capabilities cannot be achieved sufficiently with classical control approaches (Christaller, 1999; Hassabis et al., 2017).

Bio-inspired robots are usually developed using general network architectures of biological neural systems (Meyer and Guillot, 2008), synaptic plasticity (Grinke et al., 2015), correlation-based learning rule with synaptic scaling (Tetzlaff et al., 2011). Recent progresses in cognitive sciences and developmental neurobiology have promoted a new branch of robotics named “Cognitive Developmental Robotics (CDR)” (Asada et al., 2009; Asada, 2013; Min et al., 2016). Such robots behave in response to a dynamic environment by Spiking Neural Networks based controllers. CDR has emerged as a scientific field of research aiming to develop robots with abilities to effectively interact with dynamic environments and show brain-like cognitive abilities such as memory and learning. CDR has just started and its design principles and methodology have not been established (Wang et al., 2002).

To construct a software of a CDR system, a computational model of agent-environment interaction that define dynamical response of the CDR executed by a SNN with a sufficient architecture is required. Briefly, it is done as follows (Asada et al., 2009; Asada, 2013; Xu et al., 2014):

Step 1. Propose a hypothesis based on the simplification of known experimental data (so far, mainly at synaptic or network levels). The hypothesis is translated into an efficient algorithm.
Step 2. Conduct computer simulations by implementing the algorithm that is expected to be efficient for real robots from a computational complexity point of view.
Step 3. Verify the proposed hypothesis with a real robot. If hypothesis does not work, then propose a new hypothesis and go to step 1.

**Figure 1:**
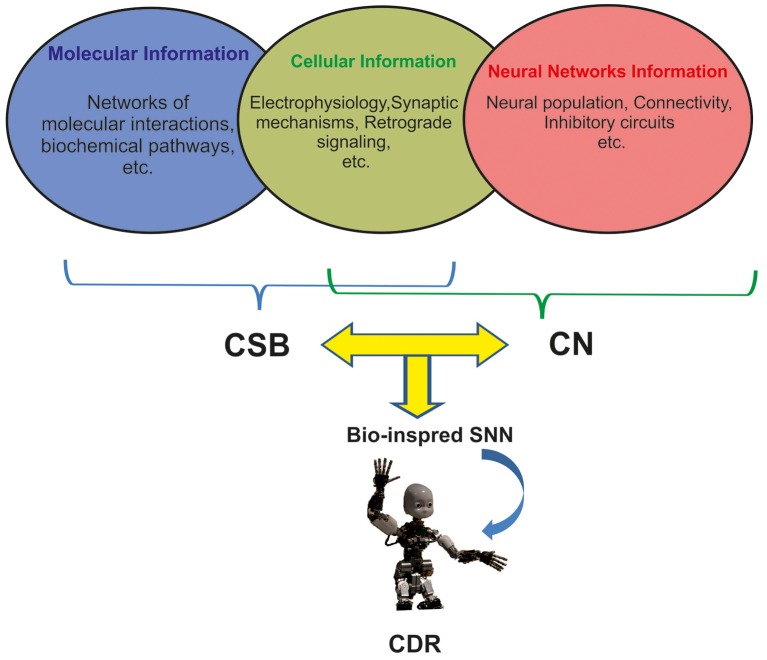
The integration of three levels of neuronal information for developing future generation of CDR. Molecular and cellular information underlying learning and memory are mainly modeled by Computational Systems Biology approaches (CSB). Neural Network information is modeled by Computational Neuroscience approaches (CN). Current Cognitive Developmental Robotics (CDR) is based on Neural Networks information; however, here we argue that the use of different levels of information by combining CSB and CN approaches in bioinspired Spiking Neural Networks (SNN) will help develop more intelligent CDR.

There are many challenges to implement natural intelligence in artificial neural systems including CDR (Xu et al., 2014). This is mainly due to the high number of neuronal populations involved in many cognitive functions, diversity of neuron types (e.g., 122 neuro types in rat hippocampus), and morphological features of neurons in sub-regions of the brains that influence different modes of spiking in neurons (burst, sparse, and normal spiking; Wheeler et al., 2015). In addition, cellular event and molecular pathways underlying neuronal changes through cognitive phenomena that take place in different time scales from seconds to days (Tetzlaff et al., 2012) are essential for investigating the cognitive abilities and to implementing them in CDR (Figure 1).

Memory and forgetting of learned knowledge from dynamic environments are essential for life by sensing certain (and important) features of the environment to react accordingly. Exploring the mechanisms of learning and memory are essential for understanding of higher cognitive functions of the brains and so to develop future CDR systems.

Current cognitive artificial systems are based on the designer's understanding of the possible mechanisms. Designer's understanding of the cognitive function can be used for controller structure in the cognitive developmental robot's brain.

## Computational systems biology of learning and memory

Many signaling pathways allow the neuron to receive, process, and respond to information from other neurons while components of different pathways interact, resulting in signaling networks. To understand learning and memory in neural systems at the systems level, the understanding of the structure and dynamics of cellular and biochemical function, rather than the characteristics of isolated parts of the brain are required (Kandel et al., 2014).

A system-level understanding of a neuronal system can be described as the network of gene interactions and biochemical pathways, as well as the mechanisms by which such interactions modulate the electrophysiological properties of neurons. Computational systems biology methods analyze biochemical signaling networks to model their role in complex biological processes (Kitano, 2002; Kotaleski and Blackwell, 2010).

To predict and investigate the properties of neural systems, computational neuroscience and computational systems biology use computational approaches to investigate more or less the same neural systems but unfortunately there has been little interaction between the two fields to explore neural mechanisms of cognition (De Schutter, 2008). Despite the similarity in aims and approaches of computational systems biology and computational neuroscience, many computational neuroscientists have little interest in molecular process of neurons (Figure 1; De Schutter, 2008).

## Cognitive developmental robotics: opportunities and challenges

The new generation of SNNs and their application in CDR research can benefit from the combination of computational systems biology and computational neuroscience approaches to develop novel algorithms for brain-like architectures with cognitive capabilities, especially memory and learning. For this purpose, a delicate simplification of complex molecular and cellular mechanisms and implementation of such computational approaches in artificial neural systems allows SNNs accurately capture human and animal behavior (Figure 1).

To consider developing brain-like architectures, we should note that biological neurons show a variety of morphological features like dendrite and axon shapes, spiking features, and different synaptic mechanisms (Wheeler et al., 2015). An important neuroscience research stream that may affect robotic studies would be the study of role and mechanisms of sparse, burst as well as normal spiking patterns in different neurons (Mendis et al., 2016).

The question is how these variations are correlated with cognitive functions and more importantly how to development them in SNNs. The implementation of biophysical and morphological features of different neurons in different parts of the brain is an essential progress toward developing of intelligent neural systems including CDR. Hence, we suggest that novel integrative approaches to develop SNNs like which integrate electrophysiological features of neural systems and algorithms from both systems biology and computational neuroscience algorithms, will allow for the development of intelligent cognitive systems (Figure 1).

Toward more intelligent CDR systems, novel inspired memory, and learning algorithms may play important roles. Sources of information are mainly molecular mechanisms of memory and learning, synaptic mechanisms and theories of large scale neural networks. Simplification of information on these fundamental capabilities of the brains and creating inspired mechanisms regarding balance of excitatory and inhibitory neurons in the neural systems are essential for developing novel CDRs using spiking neural networks (Yizhar et al., 2011).

Specially, we claim that computational systems biology based algorithms for memory and learning play fundamental roles in progress toward implementing cognitive functions in CDR. Moreover, using these algorithms in CDR systems may be used in experimental studies to design and test experiments.

Three main sources of information for CDR research using computational systems biology of learning and memory are as follows (Figure 1):

1. **Molecular information:** Modeling of molecular mechanisms of synaptic plasticity using computational systems biology approaches can provide implementation of basic memory and learning in CDR systems (Bellas et al., 2014). Long Term Potentiation (LTP), Long Term Depression (LTD), and inhibitory plasticity (Kullmann et al., 2012) are some of this molecular information. Most important challenge to achieve these goals is to demonstrate similar behavior in CDR systems while keeping time complexity of the system sufficiently. One of the examples of incorporating molecular information in artificial systems is modeling of associative learning in artificial neural networks (Smith et al., 2008). Moreover, to implement molecular mechanisms of synaptic events one can avoid information on channel expression and their biophysical activities by using simplified neuron models like Leaky Integrate Fire neuron model (LIF) that is widely used in spiking neural networks (Izhikevich, 2003).
  As an example from recent studies, SNNs have been used for the operant conditioning learning process as robot brain controllers. For this purpose, Spike Timing Dependent Plasticity (STDP) and habituation has been combined to create a network with only three neurons which implements operant conditioning (Cyr et al., 2014).
2. **Cellular information:** Non-synaptic neural communication play important role in dynamics of biological neural networks. One of these mechanisms is retrograde signaling as chemicals diffused from pre to post synaptic neurons as a consequence of synaptic stimulation (Harrington and Ginty, 2013). Recently, a bio-inspired SNN has been developed that simulates performance in first order as well as second order conditioning (Faghihi et al., 2017). The system architecture is based on *Drosophila* olfactory system and physiological roles of inhibitory neurons and simplification of systems biology of axonal retrograde signaling diffusion and function. According to a systems biology based hypothesis, retroaxonal signaling transfer information backward along axons such that strengthening of a neuron's output synapses stabilizes recent changes in the same neuron's inputs (Harris, 2008).
  In addition, while neuronal morphology is affected by environment, their role in information processing in robotic systems has not been studied. Hence, to create brain-like systems the dependency of neuronal activity and their morphology (Araya et al., 2014) should be involved in CDR research.
3. **System information:** Although neuronal types and their electrophysiology features of many sub-regions of animal's brain are remarkably known, their information processing and principles of their cognitive functions are not fully known. However, it is possible to use available information to create CDR systems. For this purpose, network size can limit the speed of the algorithms while to achieve efficient memory one needs to develop large scale neural networks. Therefore, a balance between these two parameters using simplified neuron models should be considered. Recently, a neural network named Evolved Plastic Artificial Neural Networks (EPANN; Soltoggio et al., 2017) has shown interesting capabilities to respond to sensory-output experiences. This neural system is composed of sensory unit, information processing unit and output unit (behavior unit).

As another example, inspired by anatomy and spatio-temporal learning paradigm in the hippocampus and prefrontal cortex a robot has been developed to encode sensory and temporal information (Hirel et al., 2011). This robot performs tasks requiring the behavior of the robot to integrate sensory and temporal information but it is not based on biological features of neurons in hippocampal regions. Definitely, to implement hippocampal like cognitive capabilities in artificial systems one needs to involve details of biological information at molecular, cellular and network level available in hippocampus knowledge base (Wheeler et al., 2015), thus requiring the integration of data from computational neuroscience and computational system biology (Figure 1). Hence, computational systems biology approaches can be applied to simplify such complicated biological information to integrate with computational neuroscience algorithms to modify them or present novel algorithms. Applying such algorithms in SNNs can help develop artificial systems mimicking the behavior of biological neural systems.

Regarding the complexity of memory and learning in animals' brain, computational systems biology based models can play critical role in future CDR research by integrating three mentioned levels of neuronal information underlying learning and memory (Figure 1).

Finally, we emphasize that although the complexity of computational systems biology based algorithms may limit its applications in CDR research, we believe that novel methods to develop integrated information using computational systems biology approaches can play critical role in future CDRs. Interestingly, CDRs can also provide new understanding of how cognitive functions may be developed and constructed in dynamic environment. Overcoming these challenges CDR can make progress toward more intelligent and gaining brain-like actions in the future in parallel to advances in experimental neurobiology.

## Author contributions

All authors listed have made a substantial, direct and intellectual contribution to the work, and approved it for publication.

### Conflict of interest statement

The authors declare that the research was conducted in the absence of any commercial or financial relationships that could be construed as a potential conflict of interest.
